# 

**Published:** 1889-10-19

**Authors:** 


### THE HOSPITAL

A WEEKLY INSTITUTIONAL JOURNAL OF
Science, Medicine, Nursing & Philanthropy.

SATURDAY, OCTOBER 19th, 1889.

**Hospital Doctors under Surveillance** - - - - 33
**Words of Consolation**.—The Sure Foundation - - - 34
**The Constitution of Man.** By Sir Andrew Clark - 35
**The Doctor's Pulpit.**—Physiological Candles - - - 37
**The Christian Church and the Bodily Health.** By the Rev. F. Lawrence, B.A. - - - - - - - 38
**Everybody's Page** - - - - - - - - - 39
**Notes and News** - - - - - - - - - 40
**Annotations.**—"In the Vulgar Tongue"—Sir Henry Roscoe on Pasteurism - - - - - - - - - - 42
**The Hospital Workshop.** In the Wards at the Royal Free 45
**National Pension Fund for Nurses** - - - - 47
**The Practitioner's Outlook and Retrospect:**
Medicine—Transfusion—Purpura - - - - - - 43
Surgery—Shock—Stricture - - - - - - - 43
Foreign Bodies in the Eye - - - - - 44
Jurisprudence—Changes after Death - - - - 44
Recent Research—Proteid Poisons of Jequirity—Antiseptics - - - - - - - - - - 44
New Drugs, Appliances, and Things Medical—Chancellors' Budgets and Cadbury's Cocoa - - - - 45
Ethical - - - - - - - - - - - - 45
**Cottage Industries in Scotland** - - - - - - 48
**Scraps and Gleanings** - - - - - - - - 48

**EXTRA SUPPLEMENT—THE NURSING MIRROR.**

**En Passant.**—National Pension Fund—Short Items—Miss Alcott—The Hotel Dieu—Practical Joke on a Matron—Addenbrooke's Hospital—Grimsby Hospital—Ladies as District Nurses—Exhibition of Nursing Uniforms - - xiii
**Notes from Australia** - - - - - - - xiv
**Cottage Hospital Matrons.**—I. The Training Required xv
**Everybody's Opinion.**—Registration of Midwives—The Pension Fund - - - - - - - - - - - - xv
**Appointments—Notices—Notes and Queries** - - xvi
**Vacancies—Amusements and Relaxation** - - xvi

